# Rumble Recognition: a gastrointestinal sound discrimination task to measure gastrointestinal interoception

**DOI:** 10.1038/s41598-026-59626-0

**Published:** 2026-07-18

**Authors:** Hannah S. Savage, Beth F. Longley, Hugo D. Critchley, Lydia J. Hickman, Wilson P. H. Lim, Adrian E. Yoris, Edwin S. Dalmaijer, Camilla L. Nord, Sarah N. Garfinkel

**Affiliations:** 1 Institute of Cognitive Neuroscience, University College London, Alexandra House, 17-19 Queen Square, London, WC1N 3AZ UK; 2 Department of Clinical Neuroscience, Brighton and Sussex Medical School, Brighton, UK; 3 MRC Cognition and Brain Sciences Unit, University of Cambridge, Cambridge, UK; 4 University of Bristol, Bristol, UK; 5 Department of Psychiatry, University of Cambridge, Cambridge, UK

**Keywords:** Gastroenterology, Neuroscience, Psychology, Psychology

## Abstract

Gastrointestinal interoception is the sensing, interpretation and integration of signals originating from the gut. In contrast to chemosensory aspects of gut-brain signalling, perceptual aspects of gastrointestinal viscerosensation and visceromotor control have received relatively little attention despite links to physical and mental health and wellbeing. To facilitate research into these aspects of interoception, we developed a novel gastrointestinal sound discrimination task, *Rumble Recognition*, to measure gastrointestinal interoceptive accuracy, self-beliefs, and metacognitive insight in three ecologically valid conditions; following a 3-hour fast, after drinking sparkling water, and after consuming a high-protein meal replacement shake. The discrimination task used a two-interval forced choice design to assess gastrointestinal interoceptive performance accuracy alongside self-reported confidence from which a measure of metacognitive insight was computed. This novel task was tested for proof-of-principle in a healthy sample (*n* = 45; 68% women), and we demonstrated its feasibility, tolerability, and ability to yield parameters relevant naturalistic experiences including reported experience of hunger. The task is fully automated, requires minimal, affordable equipment, and is freely available via open source code. We anticipate this task can facilitate research in this important, expanding and clinically relevant domain.

**Supplementary Information:**

The online version contains supplementary material available at https://doi.org/10.1038/s41598-026-59626-0.

## Introduction

A rumbling tummy and its accompanying abdominal feelings constitute salient behavioural cues with strong homeostatic associations, such as with hunger^1,2^ and digestion^3^, that drive behaviour change^4–6^. Gastrointestinal interoception refers to the sensing, interpretation and integration of signals originating from the stomach and small bowel^7^. In contrast to chemosensory aspects of gut–brain signalling and to cardiorespiratory aspects of interoception more generally, perceptual aspects of viscerosensation and visceromotor control have received relatively little attention. This is notable given their clear links to physical^8^ and mental health and wellbeing^5,6,9–12^. Gastrointestinal sensations contribute to what are colloquially referred to as ‘gut-feelings’ and are an often-reported aspect of emotional feelings^13^. The experience of sensations from the involuntary movement of the stomach, including upper abdominal pain from such movement is a key concern for clinical groups, including patients with Crohn’s Disease, eating disorders, anxiety or mood-related mental health conditions^1,14^. Despite clear impetus for more research, studies of gastrointestinal interoception are currently limited by the accessibility and tolerability of existing gastrointestinal interoception paradigms.

Experimental approaches to quantify individual sensitivity to gastric interoceptive sensations have typically relied on invasive methods, including balloon distention of rectum, stomach or oesophagus. These studies have focused on understanding symptoms within clinical patient groups with functional gut disorders^15–17^ or mental health conditions in which eating behaviours are of primary interest. However, poor tolerability of such methods has limited their widespread use in unaffected non-clinical or healthy individuals^2^. Furthermore, the electrical, thermal or biomechanical stimulation of viscera typically evoked by these methods is distinct from everyday gastrointestinal interoception, and/or might occur so infrequently that they would be indicative of ill-health. This has motivated the development of non-invasive behavioural tests (e.g. the 5-minute water load task^18^, the two-stage water load task^19,20^, or tasks using ingestible capsules^21,22^ that offer better tolerability and show comparable results to invasive methods, to gain valuable insights into normative facets of gastrointestinal interoception. Given the growing interest in gastric interoception, there remains a need for interoceptive tests that are relatively easy to administer and well tolerated.

The gastrointestinal tract generates sounds (borborygmi) that reflect patterns of peristaltic movement and digestive phase. Dynamic monitoring of such sounds can reveal important aspects of homeostatic function and contribute clinically to non-invasive diagnostic assessments due to this underlying relationship to changing gastrointestinal states and mechanics^3,23^. For example, bowel sounds can be used to classify individuals with irritable bowel syndrome, from those without^24^; a functional gut condition accompanied by wider alterations in interoceptive abilities and frequent co-occurring mental health conditions^25^. In the fasted state, the migrating motor complex is a rhythmic pattern of muscular contraction of the stomach and upper intestine that serves a housekeeping function, and which is associated with rumbling and growling gastric sounds. The quantity of different bowel sound types (i.e. number of single bursts or random sounds) when fasted represents an individual difference^23^, which may relate to, and/or interact with, higher-level gastrointestinal interoception perceptual sensitivities, abilities, and interpretations. Learning to perceive and interpret these gastrointestinal sounds and accompanying sensations may enhance sensitivity to interoceptive and motivational states such as sickness or hunger. Parents and caregivers contribute to how such skills develop, e.g. by asking child if they are hungry when they hear their stomach rumble, shaping associations with external contextual cues with their own beliefs about such cues^26^. Consequently, gastrointestinal states contributing to distinct physical and emotional feeling states are often idiosyncratic^27^ and, in part, socially constructed. In addition to the gurgles sometimes audible to the ear, using a stethoscope enables the detection of internal sounds that would otherwise go unheard, but which are, on a conscious or unconscious level, associated to meaningful gastric motion, visceromotor sensations, and their interpretation.

Gastrointestinal sounds are a non-invasive, easily accessible, and inexpensive to record by-product of gastrointestinal function. To our knowledge, no prior studies, or extant interoceptive tasks, have used gastrointestinal sounds to assess perceptual dimensions of gastrointestinal interoception. This study introduces a proof-of-principle novel gastrointestinal sound discrimination task, *Rumble Recognition*. This a non-invasive gastrointestinal interoception task that uses a two-interval forced choice design (2IFC) and subjective ratings to determine individuals’ gastrointestinal interoceptive accuracy, self-beliefs, and metacognitive insight. With open source code, and minimal, affordable equipment, we anticipate this task may help fill a gap within the gastrointestinal interoception literature, and facilitate research in this clinically relevant domain.

## Method

### Sample

Forty-five participants were recruited from the general population via the UCL SONA recruitment platform as well as via posters, online notice boards, and word of mouth. Participants were included if they were aged between 18 and 65 years old and had no current diagnosis of epilepsy, substance use disorder, pain disorder, stroke or diabetes; no previous or current diagnosis of schizophrenia or related conditions, or bipolar disorder; no previous cardiovascular surgery; no cardiac pacemaker or implantable cardioverter defibrillator; no bradycardia; no previous or current diagnosis of a significant cardiovascular or respiratory condition; not currently taking antipsychotics, corticosteroids, opiates, opioids or antidepressants – specifically NASSA, TCA, SARI or MAOI. Note use of SSRI or SNRIs was not an exclusion criterion if the dose had not changed in the last month. Participants were screened via an online screening form and, if eligible, were invited to attend a single 2.5-hour session at the Institute of Cognitive Neuroscience, UCL, London. All participants provided written informed consent, following a complete description of the study protocol, which was performed in accordance with the Declaration of Helsinki and approved by The UCL Human Research Ethics Committee (ICN-VW-01–08-2024).

### Gastrointestinal sound discrimination task (Rumble Recognition)

#### Design

**Fig. 1:**
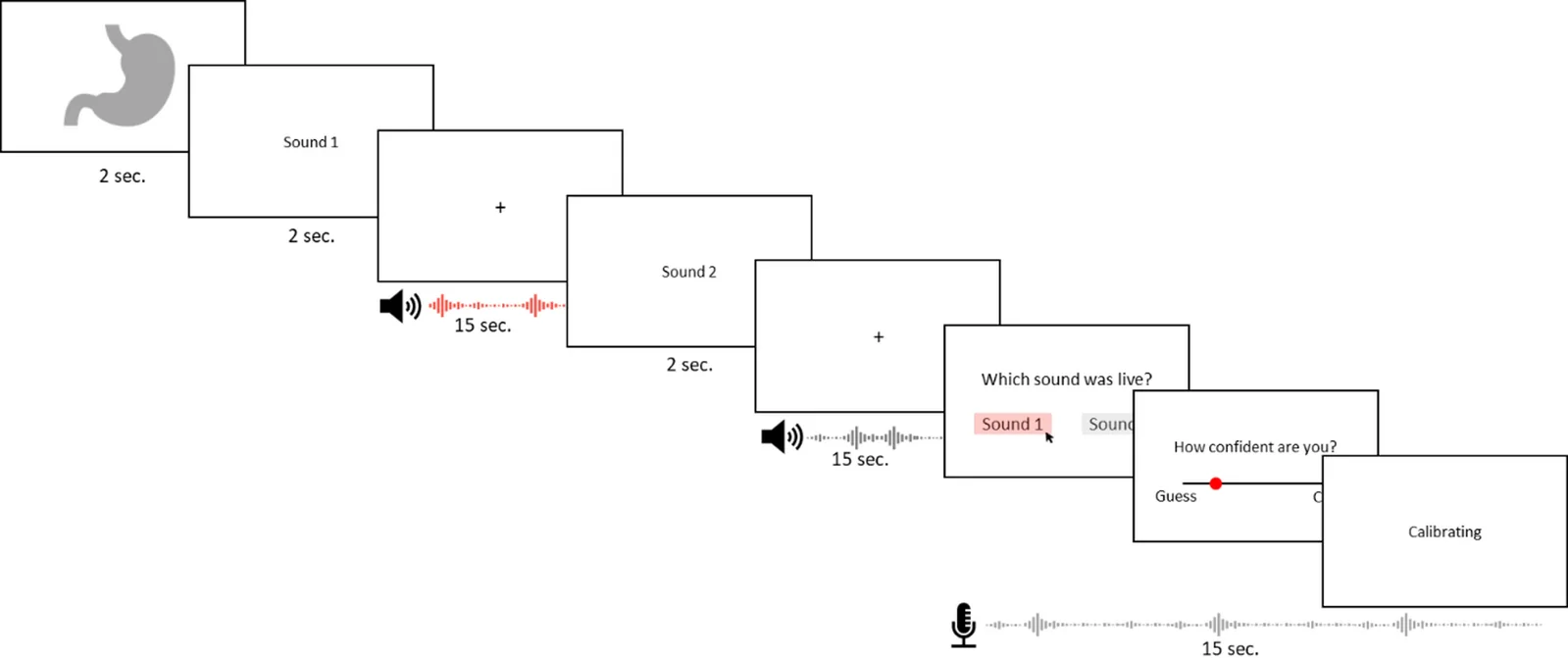
Trial structure for the gastrointestinal sound discrimination task. On each trial participants first saw an icon of a stomach on the screen. They were then presented with two 15-second sound clips: (1) one live sound (red audio), and (2) one pre-recorded sound (grey audio). After hearing both sounds, participants used the mouse to report their judgement of whether sound 1 or sound 2 was a live recording of their gastrointestinal sounds, and rated their confidence in their decision on a sliding scale from Guess (0) to Certain (100). Note, the pre-recorded sounds (grey recording) were recorded while participants were responding to subjective reports (with additional calibration time to total 15 s), so these were individualised to the participant on that day, but not a live representation of their gastrointestinal sounds. Both sounds were externally presented at comparable sound levels, with only the live recording reflecting current gastrointestinal activity.

The gastrointestinal sound discrimination task uses a two-interval forced choice design (2IFC) and subjective ratings to determine individuals’ gastrointestinal interoceptive accuracy, self-beliefs and metacognitive insight. Each trial of the task requires the participant to listen to two 15-second sound clips of their gastrointestinal sounds streamed via a digital stethoscope. One sound clip is ‘live’, i.e. played in real-time, and the alternative is pre-recorded. The participant is required to judge which of the two sounds was live (Fig. 1). Each participant completes 3 blocks, each with 10 trials; block 1 is performed in a fasted state, block 2 occurs after drinking 200 ml of sparkling water, and block 3 after drinking 200 ml of a high protein meal replacement shake. The participant provides confidence ratings for each trial judgement, and reports their self-rated experience of hunger, nausea and disgust before commencing and after each block, and their general experience at the conclusion of the task using Likert scales (see Supplementary Information).

Participants listened to all audio stimuli presented through external speakers positioned in front of the participant to ensure consistent auditory delivery across trials. Both the live and pre-recorded gastrointestinal sounds were recorded via a digital stethoscope and presented exclusively as externally delivered auditory stimuli, such that participants’ judgements were based on the experimental audio input rather than direct detection of naturally occurring bodily sounds. Both sounds were presented externally and at comparable sound levels. However, only the live recording reflected current gastrointestinal activity.

### Equipment and protocol

#### Task equipment

This task utilised a digital stethoscope (Thinklabs One Digital Stethoscope) to collect and transmit gastrointestinal sounds. Audio from the stethoscope was recorded at a sampling frequency of 1000 Hz. The stethoscope settings were as follows: Filter setting = 1 [30–500 Hz]; Volume = 0; Auto Shut Off Time Programming = Disabled. The stethoscope was connected via male audio to dual male audio cable, whereby one audio jack was plugged into the microphone jack of a USB audio adaptor plugged into the laptop running Spike2 (Cambridge Electronic Design) for physiological recording, and the other audio jack into the microphone port of the task computer (see Supplementary Information for schematic diagram). The playing and recording of gastrointestinal sounds as well as the collection of participant responses is integrated within a python-based code run in PsychoPy (v2023.1.3), on a desktop PC running Windows 11.

#### Protocol

Participants were instructed not to consume food or drink in the 3 hours prior to the start of their testing session. This task was performed first within a 2.5-hour long testing session, immediately after collecting informed consent and basic demographic information and attaching physiological recording equipment.

Participants were informed about the general aim and structure of the task (see Supplementary Information for complete instructions), and their chair was inclined to ~ 45°, before a short ‘calibration’ period. During this 30-second ‘calibration’, two 15-second sound clips were recorded while no sound was played to the participant. They then completed a single training trial, to familiarise themselves with the trial format and subjective response scale, followed by 30 trials of gastrointestinal sound discrimination.

The beginning of each trial was signalled by an icon of a stomach on the screen. Participants were then presented with two 15-second sound clips: (1) one live sound, and (2) one pre-recorded sound (Fig. 1). After hearing both sounds, participants submitted their subjective responses by using the mouse to report their judgement of whether sound 1 or sound 2 was live, and rating their confidence in their decision on a sliding scale from Guess (0) to Certain (100). The first two pre-recorded sounds were recorded during the initial calibration (these were subsequently used during the training trial and trial 1), and the remaining pre-recorded sounds were recorded while participants were submitting subjective responses (during trials 1–29). To ensure that a total of 15 seconds was recorded, if participants took less than 15 seconds to submit subjective responses, ‘Calibration’ was displayed on the screen, until the 15-second recording was completed. Recordings were always played in the next trial. Thus, both sounds (live and pre-recorded) were individualised—specific to that participant’s gastrointestinal noises on the day of testing—but only one was live, controlling for variation between subjects (or days) in gastrointestinal noise. After 10 trials (Block 1) participants quickly consumed 200 ml of sparkling water (Drink 1) and completed a further 10 trials (Block 2), then they consumed 200 ml of a high-protein meal replacement shake (Drink 2; Unsweetened Unflavoured Black Edition Huel) and completed the final 10 trials (Block 3). Participants provided confidence ratings per trial, and reported their hunger, nausea and disgust before commencing the first block, and immediately at the end of each block.

At the conclusion of the task, participants responded to a series of statements about how pleasant (‘task pleasantness’) and how hard (‘task challenge’) they found the task, their perception of the usual motility of their stomach (‘usual stomach motility’), their pre- and post-task hunger, nausea, and disgust levels (‘subjective ratings’), and how much they liked Drink 1 and Drink 2 (‘drink tolerability’) (see Supplementary Information).

## Analysis

### Stethoscope recording

#### Pre-processing

A third-order Butterworth bandpass filter (100 Hz to 1000 Hz), a 50 Hz notch filter with a quality factor of 30, and mean centring was applied to the raw stethoscope signal to isolate the relevant frequency range (100 Hz to 1000 Hz)^23,28^ and eliminate low-, high- and mains- frequency noise. All filtering operations were implemented using zero-phase filtering to avoid distortion, and implemented using scipy.signal (v1.13.1).

#### Sound composition similarity

The difficulty of this task depends on how similar the two presented sound clips (live and pre-recorded) are within each trial, and/or how similar the pre-recorded sound (played) is to the underlying true signal (not played) at that time. If both contain very few noises, the task becomes much harder, however, if one includes a prominent gurgle for example, we expect that discrimination and matching to interoceptive sensations will be easier. To quantify the ‘objective’ difficulty of each trial, we determined the similarity of the live sound and the pre-recorded sound, and of the pre-recorded sound (played) and the underlying true signal (not played) by measuring the Complexity-Invariant Distance (CID) between the two series pairs, whereby a higher CID value reflects a greater dissimilarity between the two series^29^. The CID compares the time series, considering both error (as quantified by Euclidian distance i.e. RMSE in this case), and a complexity correction factor. For example:

$$\:{CID}_{live\_recorded}=\:\sqrt{\frac{1}{n}\sum\:_{i=1}^{n}{{(Live\:Sound}_{i}-\:{Recorded\:Sound}_{i})}^{2}}\:\:\times\:\:\:\frac{\mathrm{max}(CE\left(Live\:Sound\right),\:CE(Recorded\:Sound\left)\right)}{\mathrm{min}(CE\left(Live\:Sound\right),\:CE(Recorded\:Sound\left)\right)}$$

Where CE(sound) is a complexity estimate of the time series, quantified as:

$$\:CE\left(Live\:Sound\right)=\:\sqrt{\sum\:_{i=1}^{n-1}{{(Live\:Sound}_{i}-\:{Live\:Sound}_{i+1})}^{2}}\:\:$$

### Behavioural and subjective measures

Gastrointestinal interoceptive accuracy (%) was quantified by determining the percentage of correct trials (summing the percentage correct) per condition (10 trials per block; fasted, sparkling water or high-protein meal replacement shake). We defined above-chance performance using a criterion based on binomial probability. Given the forced-choice design and the limited number of trials (10 per block; 50% chance level), we set the threshold for significantly above-chance performance at 80% (8 or more correct responses out of 10). This criterion corresponds to a binomial probability of approximately *p* = 0.05. Mean confidence was calculated by averaging the reported confidence ratings per condition. Area under the receiver operating characteristic (ROC) curve was used to quantify interoceptive metacognitive insight.

### Statistical tests

The statistical analysis employed linear mixed effects models (LMMs) to examine the relationships between various behavioural and subjective measures. Linear mixed effects models were implemented using pymer4^30^ (v0.8.2) in an environment running python v3.9.13 to determine the relationship between variables of interest, with participant always modelled as a random intercept.

i) Task experience and tolerability.

To evaluate task experience and tolerability, mean ratings were calculated by averaging the reported ratings across participants. A paired t-test was used to compare ratings of the sparkling water to the high-protein meal replacement shake.

ii) Subjective ratings (Hunger, Nausea, Disgust).

LMMs were used to assess changes in subjective ratings over the course of the experiment:

- Hunger Rating ∼ Instance + (1 | Participant).
- Nausea Rating ∼ Instance + (1 | Participant).
- Disgust Rating ∼ Instance + (1 | Participant).

Where appropriate, follow up t-tests were performed and Bonferroni-corrected for multiple comparisons.

iii) Interoceptive accuracy, confidence, and metacognitive insight.

We investigated the main effects of condition (fasted, sparkling water or high-protein meal replacement shake) on interoceptive accuracy, confidence, and metacognitive insight (AUC). Separate LMMs were run for each outcome variable:

- Accuracy ∼ Condition + (1 | Participant).
- Confidence ∼ Condition + (1 | Participant).
- AUC ∼ Condition + (1 | Participant).

We also further explored the relationship between confidence and accuracy, with an LMM testing for interactions with condition (fasted, sparkling water or high-protein meal replacement shake):

- Confidence ∼ Accuracy + Condition + Accuracy × Condition + (1 | Participant).

iv) Influence of bowel sound composition - Trial-level analysis.

A generalized linear mixed model (GLMM) with a binomial distribution was used to analyse the influence of bowel sound composition on accuracy at the trial level:

- Accuracy per trial (GLMM, Binomial) ∼ Condition + Rec_Live + Rec_NotPlay + Rec_Live × Rec_NotPlay + (1 | Participant).

The influence of bowel sound composition on confidence was analysed at the trial level using an LMM:

- Confidence ∼ Condition + Rec_Live + Rec_NotPlay + Rec_Live × Rec_NotPlay + (1 | Participant).

The influence of bowel sound composition on the relationship between confidence and accuracy at the trial level was also modelled:

- Confidence ∼ Accuracy per trial + Condition + Rec_Live + Rec_NotPlay + Rec_Live × Rec_NotPlay + (1 | Participant).

xxiiv) Influence of hunger on task performance.

We further investigated the impact of hunger on accuracy, confidence, and AUC. To achieve this, we included the average of pre- and post- condition hunger ratings per block (referred to as ‘average hunger’) as a measure of generally how hungry a participant was during that condition (fasted, sparkling water or high-protein meal replacement shake), or the magnitude of change in hunger across the block (referred to as ‘hunger change’) as predictors, testing for interactions with condition:

- Accuracy ∼ Condition + Average Hunger + Average Hunger × Condition + (1 | Participant).
- Accuracy ∼ Condition + Hunger Change + Hunger Change × Condition + (1 | Participant).
- Confidence ∼ Condition + Average Hunger + Average Hunger × Condition + (1 | Participant).
- Confidence ∼ Condition + Hunger Change + Hunger Change × Condition + (1 | Participant).
- AUC ∼ Condition + Average Hunger + Average Hunger × Condition + (1 | Participant).
- AUC ∼ Condition + Hunger Change + Hunger Change × Condition + (1 | Participant).

**Note**: This analysis was not performed for nausea or disgust due to non-significant changes in subjective ratings over the course of the experiment.

vi) Test of trial effect.

We additionally tested whether there was a trial effect on interoceptive accuracy and confidence. Separate LMMs were run for each outcome variable:

- Accuracy ∼ Trial + (1 | Participant).
- Confidence ∼ Trial + (1 | Participant).

### Data and code availability

Data is freely available on Zenodo (doi: https://doi.org/10.5281/zenodo.16900962), or by contacting the corresponding author. All code to run the task is available at: https://github.com/TheEmBodyStudy/RumbleRecognition, where you will also find installation instructions, links to the equipment used, and comprehensive instructions to run the task. Code to perform the analysis in this manuscript will also be made available.

## Results

### Task experience, tolerability and subjective ratings

**Fig. 2:**
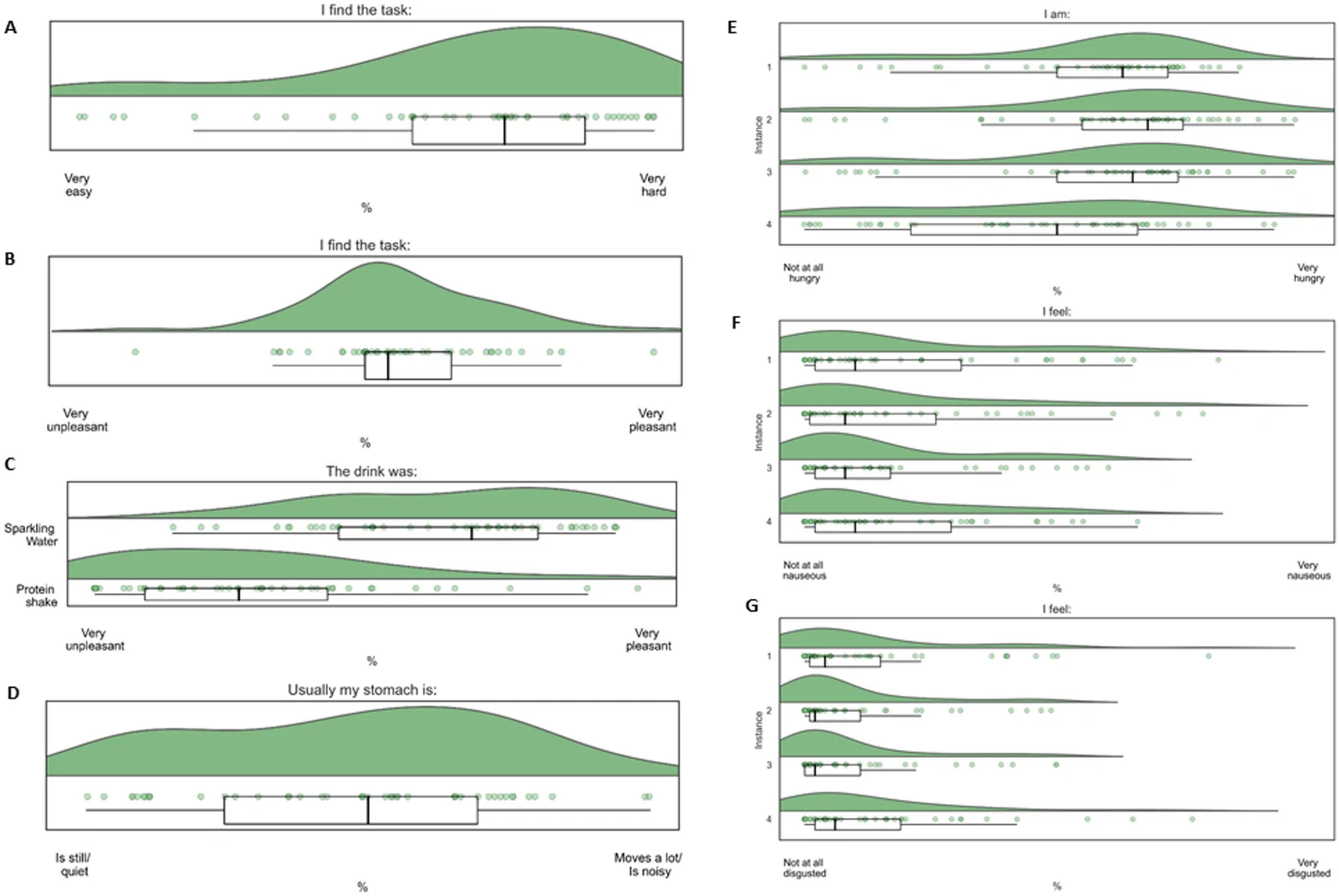
Participant task experience and tolerability ratings. End of task subjective reports of **(A)** task challenge, **(B)** task pleasantness, **(C)** drink tolerability, and **(D)** usual stomach motility. Ratings of **(E)** hunger, **(F)** nausea, and **(G)** disgust at four time points; prior to the task beginning and at the conclusion of each condition.

Overall, participants found the task fairly challenging (mean rating: 67.57 ± 27.70; Scale: Very easy = 0 to Very hard = 100, Fig. 2A) and generally neutral-somewhat pleasant to complete (mean rating: 56.04 ± 15.43; Scale: Very unpleasant = 0 to Very pleasant = 100, Fig. 2B). Sparkling water was significantly more pleasant to drink than the high-protein meal replacement shake (sparkling water mean rating: 62.88 ± 22.19; high-protein meal replacement shake mean rating: 28.22 ± 24.35; Scale: Very unpleasant = 0 to Very pleasant = 100; Paired t-test T statistic = −7.028, *p* < 0.001, Fig. 2C). On a scale from Quiet (0) to Moves a lot/Noisy (100), participants said their stomachs were usually somewhat noisy (mean rating = 48.13 ± 26.78, Fig. 2D).

Participants reported themselves as somewhat hungry when the task began (57.08 ± 21.36) and there was a significant effect of time on subsequent hunger ratings (β = −3.76, *p* < 0.001), which follow-up Bonferroni-corrected paired t-tests found was due to the rating after the high-protein meal replacement shake being significantly lower than all previous time points (pre-task vs. post high-protein meal replacement shake p_corrected_ = 0.003; post fasting vs. post high-protein meal replacement shake p_corrected_ < 0.0001; post sparkling water vs. post high-protein meal replacement shake p_corrected_ < 0.0001, Fig. 2E). Nausea and disgust scores did not significantly change during the task from their initial averages of 19.26 ± 22.48, and 12.71 ± 18.31, respectively (all *p* > 0.1, Fig. 2F and G).

### Gastrointestinal interoceptive accuracy

**Fig. 3:**
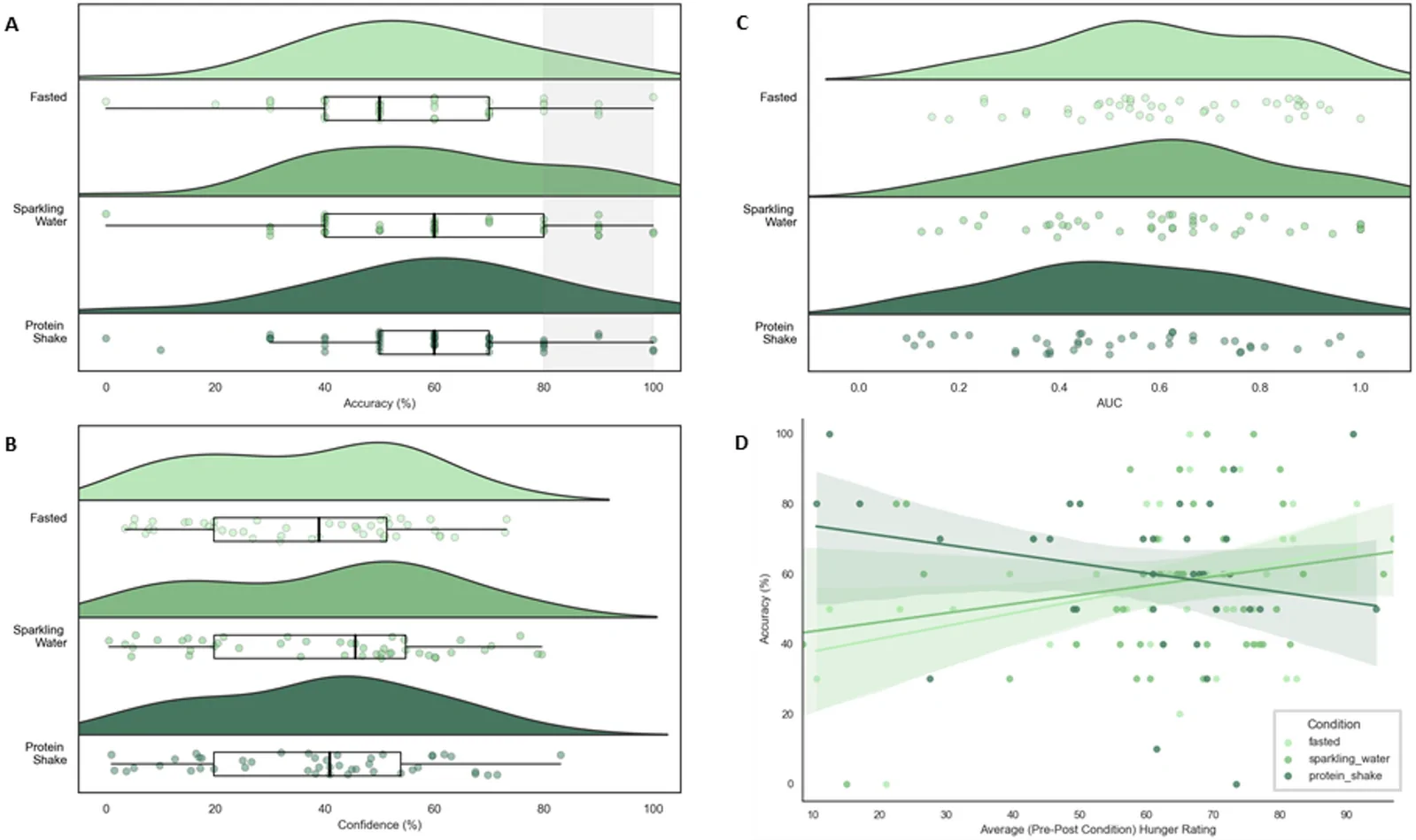
Performance across interoceptive domains. **(A)** Accuracy identifying the live sound, per drink condition. **(B)** Mean confidence rating, per drink condition. **(C)** Interoceptive insight (quantified as area under ROC curve (AUC)), per drink condition. **(D)** Influence of hunger on task performance.

Participants’ average accuracy during the fasted condition was 55.77 ± 20.39% (95% CI [49.81, 61.73]). A linear mixed model found drinking sparkling water non-significantly increased accuracy by 3.11% (β = 3.11, *p* = 0.445; mean = 58.88 ± 22.38% (95% CI [52.34, 65.42])), and the high-protein meal replacement shake by 4.0% (β = 4.00, *p* = 0.326; mean = 59.77 ± 22.20% (95% CI [53.28, 66.26])), when compared to fasted, as shown in Fig. 3A. There was a significant effect of participant (*p* < 0.001). A contrast test found there was no significant difference between the sparkling water and high-protein meal replacement shake conditions (*p* = 0.826). During the fasted condition, 17.78% of the sample had accuracy levels equal to, or greater than 80%, which increased to 26.67% and 24.44% for sparkling water and the high-protein meal replacement shake conditions respectively. A check for an effect of trial number on accuracy (i.e. learning effects) was not significant (β = 0.002, *p* = 0.161).

### Gastrointestinal interoceptive confidence

Participants’ average confidence during the fasted condition was 36.38 ± 19.98% (95% CI [30.54, 42.22]). A linear mixed model found drinking sparkling water marginally, but not-significantly increased reported confidence by 3.69% (β = 3.698, *p* = 0.062; mean = 40.08 ± 22.49% (95% CI [33.51, 46.66])), and non-significantly increased reported confidence after drinking the high-protein meal replacement shake by 2.35% (β = 2.358, *p* = 0.232; mean = 38.74 ± 20.85% (95% CI [32.65, 44.84])), when compared to fasted, as illustrated in Fig. 3B. There was a significant effect of participant (*p* < 0.001). Overall, there was a small (β = 0.14) but significant effect of trial on confidence ratings (*p* = 0.017).

### Gastrointestinal interoceptive insight

Overall, an LMM found self-reported confidence was significantly related to increased accuracy (β = 0.254, *p* < 0.0001). An LMM found that interoceptive insight, as quantified by the area under the ROC curve (AUC) and excluding participants for whom this could not be calculated in one or more conditions (*n* = 7), was not significantly different from fasted (mean = 0.58 ± 0.22; sparkling water: mean = 0.59 ± 0.22, β = 0.016, *p* = 0.747; high-protein meal replacement shake: mean = 0.52 ± 0.24, β = −0.056, *p* = 0.249), as shown in Fig. 3C. There was a significant effect of participant (*p* < 0.001). Mean insight for the task overall, was 0.56 ± 0.23.

### Influence of bowel sound composition (i.e. afferent signal) on task performance

The difficulty of this task is modulated by the similarity of the live and pre-recorded sounds (rec_live), as well as the comparison between the pre-recorded sound and what is occurring at the time that is played (rec_recplay). Staged linear mixed models found no influence of similarity for either of these sound-pairs, or their interaction on accuracy (rec_live: β = 0.048, *p* = 0.054, rec_recplay: β = 0.002, *p* = 0.595, rec_live*rec_recplay: β = −0.001, *p* = 0.867). They further showed the more dissimilar (a higher CID) the live and pre-recorded sounds (rec_live) the greater participants rated their confidence in their decision (β = 4.479, *p* < 0.001), and the effect of the sparkling water condition was now significant (β = 3.547, *p* = 0.004). Interestingly, when accuracy was included in the model, both accuracy (β = 6.54, *p* < 0.001) and the dissimilarity between the live and pre-recorded sound (β = 4.159, *p* < 0.001) were significant predictors of confidence. The effect of the sparkling water condition remained significant (β = 3.354, *p* = 0.006). In all models there was a significant effect of participant.

### Influence of hunger on task performance

We found that average hunger significantly interacted with condition to influence accuracy. Participants who were hungrier at the start of the task, generally performed better (main effect of average hunger (β = 0.382, *p* = 0.004)), and the significant increase in accuracy performance during the high-protein meal replacement shake condition (β = 42.52, *p* = 0.004), was most pronounced for individuals who were the least hungry by this stage of the experiment (β = −0.639, *p* = 0.006, Fig. 3D). Average hunger significantly increased confidence (β = 0.391, *p* = 0.040), but there were no hunger x condition interactions (all *p* > 0.1), and interoceptive insight was not related to average hunger (all *p* > 0.069). No significant effects of hunger, or its interaction with condition emerged when using hunger change scores (Accuracy: all *p* > 0.5; Confidence: all *p* > 0.4; Insight: all *p* > 0.3).

## Discussion

This paper introduces a proof-of-principle novel gastrointestinal sound discrimination task (*Rumble Recognition)* that is well tolerated and easy to administer, and has the potential to capture individual differences in gastrointestinal interoceptive accuracy, self-beliefs and metacognitive abilities. This task can be easily administered and addresses a gap in current gastrointestinal interoception research.

Our findings extend prior studies of gastrointestinal interoceptive illusions, suggesting that participants align their internal perceptions with the auditory input, rather than adjusting their interpretation of auditory cues based on interoceptive sensations^26^. We did not observe an influence of the discrepancy between the pre-recorded sound and the concurrent gastrointestinal sounds (not played) on accuracy or confidence ratings. We take this to suggest that the matching of the sounds to felt sensations (with sound interpreted as ground truth) might be the main mechanism by which participants complete this task, rather than the matching of felt sensations to the presence or absence of a sound (with sensations as ground truth). This could be because the auditory input offers a more immediate and precise reference point for matching than the more diffuse or delayed interoceptive sensations^31^. A previous study reported that playing participants gastrointestinal rumbles caused many to mis-localise the sounds as coming from their own stomach. This effect increased reported desire to eat depicted food, particularly if they believed the stomach rumble meant hunger^26^. In this context, our observations reinforce the notion that while gastrointestinal interoceptive accuracy closely relates to pre-attentive viscerosensory signals from the gut (linked to allostasis), the integration and higher order appraisal of exteroceptive information about gastrointestinal states (i.e. what does it mean when you *hear* your stomach rumble in certain contexts) is influenced by prior socially-mediated associative learning of the meaning of this signal^27^. This aspect is reflected in self-beliefs as captured by the in-task confidence ratings.

This task accesses a key function of gastrointestinal interoception: informing hunger state. This is evident in the observed relationship between gastrointestinal interoceptive accuracy and hunger ratings. The hungrier participants were when fasted, the more accurate they were on the task, and later, the individuals who were the least hungry by the end of the task were more accurate than those who remained hungry. These findings indicate that individuals who are more accurate at detecting their gastrointestinal state (stomach is empty, stretched, or activated by nutritional components) may be more influenced unconsciously by these signals. This subconscious influence is further evidenced by studies that have found stomach acidity^32^ and gastric motility^33^ are related to the intensity of emotions including disgust, and work in rodents that showed increased activation of vagal afferents, usually stimulated by stretch and nutrient-derived signals, was related to an increase in anxiety-like symptoms^4^. Relatedly, Poovey and Rancourt^34^ found individuals who reported greater visceral sensitivity and responsiveness to hunger cues were those who were more likely to engage in pathological emotional eating (i.e. binge eating and/or purging), which others suggest could be mediated by cortisol-related mechanisms^35^. Thus, it would be interesting for future studies to use this task in clinical groups where gastrointestinal interoception (and higher-order appraisals of these signals^36)^ may be altered, including those with anorexia nervosa^37,38^ and functional dyspeptic symptoms^11,15,18^. If accuracy is indeed heightened in physiologically meaningful states, as our results show, it would be critical to ascertain the directionality of this effect to potentially mitigate the gastrointestinal-related symptoms that are commonly reported^39^ in psychologically heightened states, such as in states of stress or anxiety. Given this relationship to hunger was not observed for confidence or insight in our study, it may be that this effect predominantly works pre-consciously to optimise allostatic behaviour (e.g. eating in anticipation of nutritional need). This might provide a window into understanding the origins of gastrointestinal symptoms (and more generally, the role of brain-to-gut communication) in mental health conditions, a priority for interoceptive research both scientifically^9^ and in terms of patient priorities^14^.

This *Rumble Recognition* study used gastrointestinal sounds recorded in three naturalistic states; fasted, after consumption of sparkling water, and after consumption of a high-protein meal replacement shake. In the fasted state, the migrating myoelectrical/motor complex^40^, a very slow cyclical pattern (~ 60–110 min per cycle) that occurs during the interdigestive state of humans and other animals, regulates contractions of the stomach and duodenum. The hollow rumbles that we hear and learn during development to associate with hunger^27^ are most likely to occur during phase III of this cycle. Two affordable and accessible interventions (ingestion of 200 ml of sparking water and 200 ml of a high-protein meal replacement shake) were used in the task to modify gastrointestinal distention and motility, inducing changes in stomach signals and sensations that are commonly experienced during everyday life. Activation of the trigeminal nerve innervating the tongue by the carbonated water stimulates the dorsal vagus nucleus in the brainstem, promoting a change in upper gastrointestinal motility^41^. Sparkling water consumed on a fasted stomach should rapidly pass through to the duodenum where dissolved carbon dioxide is transformed to bicarbonate and/or released in gaseous form^42^. This process results in a build-up of luminal gas, increasing gastrointestinal pressure. In larger volumes than used in this study (> 300 ml) gastrointestinal pressure alone can lead to sensations of satiation^42^. The subsequent ingestion of a high-protein meal replacement shake elicits the mucosal sensing of changed luminal content, including osmolarity, presence of lipids and products of carbohydrate digestion, and evokes mucosal biomechanical stimulation contribute to the sensation of intestinal contents and further stimulates digestive processes. This disrupts the migrating myoelectrical/motor complex and instigates peristaltic digestive movements. The total amount of liquids ingested after the second drink was 400 ml, which is around the volume many individuals in the two-stage water load test report satiation^19^.

In its current proof-of-principle structure, this task balances three experimental conditions, participant burden and overall duration. As a result, there are ten trials per condition which is arguably a sub-optimal number of trials when balancing both reliability and power^43^. At the group level, average interoceptive accuracy was close to chance level (55.77%, 58.88%, 59.77% in the fasted, sparkling water, and high-protein meal replacement shake conditions, respectively), with substantial variability across participants. While this may initially appear to reflect poor performance, such variability is consistent with prior interoception research, where group-level accuracy often approximates chance despite meaningful individual differences. Importantly, a subset of participants consistently performed above chance (17.78%, 26.67%, and 24.44% of participants in the fasted, sparkling water, and high-protein meal replacement shake conditions, respectively). In addition, accuracy varied with physiological state (i.e. hunger), indicating that the task is sensitive to state-dependent differences in gastrointestinal interoceptive processing. Thus, the utility of the task lies not in elevated mean accuracy, but in its ability to capture meaningful variability in interoceptive sensitivity under controlled conditions. This variability in performance enables the investigation of how individual differences in gastrointestinal interoception may relate to mental health symptoms such as anxiety or depression.

Although, task performance was not compared to established measures of gastrointestinal interoception, the current study primarily aimed to evaluate the feasibility and tolerability of Rumble Recognition in a representative general population sample, as well as to investigate how interoceptive dimensions (e.g. accuracy, confidence, insight) vary across three ecologically valid physiological conditions, and to examine how these measures interact with physiological states such as hunger. However, future research directly comparing Rumble Recognition to established measures would enable a better understanding of how distinct aspects of gastrointestinal interoception (e.g. influence of hunger on interoceptive accuracy, perception of satiety, perceptual accuracy of the detection of ingested vibrating capsules) might differentially relate to specific emotions (e.g. disgust), as well as how this might differ across populations (e.g. in individuals with eating disorders).

A further limitation of the present study is that the sound clips were presented through external speakers, which may have allowed audible bodily sounds to inform recognition decisions. The use of over-ear headphones, instead of external speakers, may reduce auditory interferences and enhance task sensitivity. We recommend that future studies employ over-ear headphones to help ensure that participants are reliant on interoceptive information to complete the task.

In our study, individuals with eating disorders and/or gastrointestinal conditions were not excluded in order to preserve ecological validity and to capture natural variation in gastrointestinal interoceptive ability within the general population. Future studies may compare performance across clinical and non-clinical groups to assess potential differences in gastrointestinal interoceptive processing. This task is flexible for future users in terms of the number of conditions that can be used and the number of trials that can be included, such that it can be tailored to suit specific experimental questions and study population demands. For example, researchers could investigate the influence of gut acidity/pH (measurable using ingestible capsules^32^ and modifiable using antacids), or gastric myoelectrical activity (which can be normalised using anti-nausea medications^33^) on domains of gastrointestinal interoception.

Importantly, this study shows that *Rumble Recognition*, our novel gastrointestinal sound discrimination task, is well tolerated, easy to administer, and captures individual differences in gastrointestinal interoceptive accuracy, self-reports, and metacognitive abilities. This task requires minimal equipment and has freely downloadable code, making it an affordable and easily implementable solution to an existing gap in the gastrointestinal interoception literature and methods.

## Supplementary Information

Below is the link to the electronic supplementary material.

Supplementary Material 1

## Data Availability

Data is freely available on Zenodo (doi: 10.5281/zenodo.16900962), or by contacting the corresponding author. All code to run the task is available at: https://github.com/TheEmBodyStudy/RumbleRecognition, where you will also find installation instructions, links to the equipment used, and comprehensive instructions to run the task. Code to perform the analysis in this manuscript will also be made available.

## References

[1] Stevenson, R. J. et al. Interoceptive hunger, eating attitudes and beliefs. *Front. Psychol.* **14**, 1148413 (2023). 10.3389/fpsyg.2023.1148413 PMC10160651 37151322

[2] Whitehead, W. E. & Drescher, V. M. Perception of Gastric Contractions and Self-Control of Gastric Motility. *Psychophysiology* **17**, 552–558. 10.1111/j.1469-8986.1980.tb02296.x (1980). 7443922

[3] Cannon, W. B. Auscultation of the rhythmic sounds produced by the stomach and intestines. *Am. J. Physiology-Legacy Content*. **14**, 339–353 (1905).

[4] Krieger, J. P. et al. Neural Pathway for Gut Feelings: Vagal Interoceptive Feedback From the Gastrointestinal Tract Is a Critical Modulator of Anxiety-like Behavior. *Biol. Psychiatry*. **92**, 709–721. 10.1016/j.biopsych.2022.04.020 (2022). PMC11438499 35965105

[5] Whitehead, W. E. in *in Psychophysiology of the Gastrointestinal Tract: Experimental and Clinical Applications*. 333–350 (eds Rupert, Hölzl & Whitehead, W. E.) (Springer US, 1983).

[6] Prescott, S. L. & Liberles, S. D. Internal senses of the vagus nerve. *Neuron* **110**, 579–599 (2022). 10.1016/j.neuron.2021.12.020 PMC8857038 35051375

[7] Suksasilp, C. & Garfinkel, S. N. Towards a comprehensive assessment of interoception in a multi-dimensional framework. *Biological Psychology* 108262. 10.1016/j.biopsycho.2022.108262 (2022). 35026353

[8] Brock, C., Arendt-Nielsen, L., Wilder-Smith, O. & Drewes, A. M. Sensory testing of the human gastrointestinal tract. *World J. Gastroenterol.* **15**, 151–159. 10.3748/wjg.15.151 (2009). PMC2653306 19132764

[9] Nord, C. L. & Garfinkel, S. N. Interoceptive pathways to understand and treat mental health conditions. *Trends Cogn. Sci.* **26**, 499–513. 10.1016/j.tics.2022.03.004 (2022). 35466044

[10] Banellis, L., Rebollo, I., Nikolova, N. & Allen, M. Increased stomach-brain coupling indexes a dimensional signature of negative mental health symptoms. *bioRxiv* 2024.2006.2005.597517. 10.1101/2024.06.05.597517 (2024).

[11] Norton, G. R., Norton, P. J., Asmundson, G. J. G., Thompson, L. A. & Larsen, D. K. Neurotic butterflies in my stomach: The role of anxiety, anxiety sensitivity and depression in functional gastrointestinal disorders. *J. Psychosom. Res.* **47**, 233–240. 10.1016/S0022-3999(99)00032-X (1999). 10576472

[12] Ahlich, E., Poovey, K. & Rancourt, D. Examination of the two-step water load test as a measure of gastric interoception and associations with eating and weight/shape concerns in a nonclinical sample. *International Journal of Eating Disorders* **56**, 1432–1443. 10.1002/eat.23964 (2023). 37072377

[13] Nummenmaa, L., Glerean, E., Hari, R. & Hietanen, J. K. Bodily maps of emotions. *Proceedings of the National Academy of Sciences* **111**, 646–651. 10.1073/pnas.1321664111 (2014). PMC3896150 24379370

[14] Hickman, L. J. et al. Breaking through the mind-body divide: Patient priorities for interoception research. *eClinicalMedicine*10.1016/j.eclinm.2025.103183 (2025). PMC11999071 40235945

[15] Chen, C. L., Lin, H. H., Chen, M. C. & Huang, L. C. Dyspeptic symptoms and water load test in patients with functional dyspepsia and reflux disease. *Scand J Gastroenterol* **40**, 28–32. 10.1080/00365520410009483 (2005). 15841711

[16] Vandenberghe, J. et al. Regional cerebral blood flow during gastric balloon distention in functional dyspepsia. *Gastroenterology* **132**, 1684–1693. 10.1053/j.gastro.2007.03.037 (2007). 17484866

[17] Yuan, Y. Z. Functional brain imaging in irritable bowel syndrome with rectal balloon-distention by using fMRI. *World J Gastroenterol* **9**, 1356–1360. 10.3748/wjg.v9.i6.1356 (2003). PMC4611816 12800256

[18] Jones, M. P., Hoffman, S., Shah, D., Patel, K. & Ebert, C. C. The water load test: observations from healthy controls and patients with functional dyspepsia. *American Journal of Physiology-Gastrointestinal and Liver Physiology* **284**, G896–G904. 10.1152/ajpgi.00361.2002 (2003). 12529263

[19] Van Dyck, Z. et al. The water load test as a measure of gastric interoception: Development of a two-stage protocol and application to a healthy female population. *PLoS One* **11**, e0163574 (2016). 10.1371/journal.pone.0163574 PMC5033375 27657528

[20] Davey, S., Bell, E. & Halberstadt, J. Adapting a two-stage water load test to measure gastric sensitivity over time. *Physiol. Behav.* **253**, 113856. 10.1016/j.physbeh.2022.113856 (2022). 35623414

[21] Mayeli, A. et al. Parieto-occipital ERP indicators of gut mechanosensation in humans. *Nature communications* **14**, 3398. 10.1038/s41467-023-39058-4 (2023). PMC10264354 37311748

[22] Smith, R. et al. Gut inference: A computational modelling approach. *Biol. Psychol.* **164**, 108152. 10.1016/j.biopsycho.2021.108152 (2021). PMC8429276 34311031

[23] Du, X., Allwood, G., Webberley, K. M., Osseiran, A. & Marshall, B. J. Bowel sounds identification and migrating motor complex detection with low-cost piezoelectric acoustic sensing device. *Sensors*10.3390/s18124240 (2018). PMC6308494 30513934

[24] Du, X. et al. Noninvasive diagnosis of irritable bowel syndrome via bowel sound features: Proof of concept. *Clinical and Translational Gastroenterology*10.14309/ctg.0000000000000017 (2019). PMC6602778 30908308

[25] Fournier, A. et al. Interoceptive abilities in inflammatory bowel diseases and irritable bowel syndrome. *Front. Psychiatry*. **11**, 229 (2020). 10.3389/fpsyt.2020.00229 PMC7142209 32300314

[26] Stevenson, R. J. et al. Psychological induction of interoceptive hunger cues and their effect on food desire. *Appetite.* **206**, 10.1016/j.appet.2025.107855 (2025). 39798932

[27] Stevenson, R. J. et al. The development of interoceptive hunger signals. *Dev. Psychobiol.* **65**, e22374. 10.1002/dev.22374 (2023). PMC10107831 36811369

[28] Du, X. et al. A mathematical model of bowel sound generation. *J. Acoust. Soc. Am.* **144**, El485. 10.1121/1.5080528 (2018). 30599659

[29] Batista, G. E. A. P. A., Keogh, E. J., Tataw, O. M. & de Souza, V. M. A. CID: an efficient complexity-invariant distance for time series. *Data Min. Knowl. Disc.* **28**, 634–669. 10.1007/s10618-013-0312-3 (2014).

[30] Jolly, E. Pymer4: Connecting R and Python for linear mixed modeling. *J. Open. Source Softw.* **3**, 862 (2018).

[31] Barrett, L. F. & Simmons, W. K. Interoceptive predictions in the brain. *Nat. Rev. Neurosci.* **16**, 419–429. 10.1038/nrn3950 (2015). PMC4731102 26016744

[32] Porciello, G., Monti, A., Panasiti, M. S. & Aglioti, S. M. Ingestible pills reveal gastric correlates of emotions. *eLife* **13**, e85567. 10.7554/eLife.85567 (2024). PMC11149936 38831694

[33] Nord, C. L., Dalmaijer, E. S., Armstrong, T., Baker, K. & Dalgleish, T. A Causal Role for Gastric Rhythm in Human Disgust Avoidance. *Curr. Biol.* **31**, 629–634e623. 10.1016/j.cub.2020.10.087 (2021). PMC7883304 33238154

[34] Poovey, K. & Rancourt, D. Visceral sensitivity, hunger responsiveness, and satiety responsiveness: Associations between facets of gastrointestinal interoception and disordered eating profiles in an undergraduate sample. *Appetite* **196**, 107252. 10.1016/j.appet.2024.107252 (2024). 38355050

[35] Gluck, M. E., Geliebter, A., Hung, J. & Yahav, E. Cortisol, hunger, and desire to binge eat following a cold stress test in obese women with binge eating disorder. *Biopsychosoc. Sci. Med.* **66**, 876–881 (2004). 10.1097/01.psy.0000143637.63508.47 15564352

[36] Savage, H. S. & Garfinkel, S. N. Making Sense of Sensation: A model of Interoceptive Attribution and Appraisal with Clinical applications. *PsyArXiv* (2025).

[37] Khalsa, S. S., Berner, L. A. & Anderson, L. M. Gastrointestinal interoception in eating disorders: charting a new path. *Curr. psychiatry Rep.* **24**, 47–60 (2022). 10.1007/s11920-022-01318-3 PMC8898253 35061138

[38] Khalsa, S. S. et al. Altered interoceptive awareness in anorexia nervosa: Effects of meal anticipation, consumption and bodily arousal. *Int. J. Eat. Disord.* **48**, 889–897. 10.1002/eat.22387 (2015). PMC4898968 25712775

[39] Holtmann, G. & Enck, P. Stress and Gastrointestinal Motility in Humans: A Review of the Literature. *Neurogastroenterology Motil.* **3**, 245–254. 10.1111/j.1365-2982.1991.tb00068.x (1991).

[40] Deloose, E., Janssen, P., Depoortere, I. & Tack, J. The migrating motor complex: control mechanisms and its role in health and disease. *Nat. reviews Gastroenterol. Hepatol.* **9**, 271–285 (2012). 10.1038/nrgastro.2012.57 22450306

[41] Dessirier, J. M., Simons, C. T., Carstens, M. I., O’Mahony, M. & Carstens, E. Psychophysical and neurobiological evidence that the oral sensation elicited by carbonated water is of chemogenic origin. *Chem. Senses*. **25**, 277–284. 10.1093/chemse/25.3.277 (2000). 10866986

[42] Cuomo, R., Sarnelli, G., Savarese, M. F. & Buyckx, M. Carbonated beverages and gastrointestinal system: between myth and reality. *Nutr. Metab. Cardiovasc. Dis.* **19**, 683–689. 10.1016/j.numecd.2009.03.020 (2009). 19502016

[43] Kleckner, I. R., Wormwood, J. B., Simmons, W. K., Barrett, L. F. & Quigley, K. S. Methodological recommendations for a heartbeat detection-based measure of interoceptive sensitivity. *Psychophysiology* **52**, 1432–1440. 10.1111/psyp.12503 (2015). PMC4821012 26265009

