# Supplementary material for: Rumble Recognition: a gastrointestinal sound discrimination task to measure gastrointestinal interoception
Source: Sci Rep. 2026 Jul 18;16:32812. doi: 10.1038/s41598-026-59626-0 (PMC13677432; doi:10.1038/s41598-026-59626-0)
Supplement: Supplementary file 1 [file 41598_2026_59626_MOESM1_ESM.docx]

**Rumble Recognition: a gastric sound discrimination task to measure gastric interoception**

**SUPPLEMENTARY INFORMATION**

***Participant instructions***

In this task we are interested to know how well people can detect sensations from their stomach. You will hear two sounds - in one of them we will play your stomach sounds to you live, and in the other we will play pre-recorded sounds from your stomach. Your task is to figure out which of the sounds, sound 1 or 2, is live.

You will see an icon of a stomach on the screen as your signal that the sounds are about to begin. Once they have finished, you will be asked to submit your answer by clicking the button using the mouse on sound 1 or sound 2. Then, you need to indicate how confident you are in your decision using a sliding scale from 'Guess’ to ‘Certain’. First move the mouse to move the ball, and then click to lock it into place.

You will do this 10 times, then you will drink Drink 1 (fizzy water) and do this another 10 times, then you drink Drink 2 (Huel) and do this a final 10 times. The whole task takes about 30 minutes. Before you start, throughout, and at the end there are some questions about how you feel and how you found the task which you answer using the same type of sliding scale as before.

***In task questions***

Prior to commencing the task, and at the conclusion of each block task participants responded to the following statements:

I am – [Not at all hungry - Very hungry]
I feel – [Not at all nauseous - Very nauseous]
I feel – [Not at all disgusted - Very disgusted]

***Post task questions***

At the conclusion of the task participants responded to the following statements:

I find the task – [Very unpleasant - Very pleasant]
I find the task – [Very easy – very hard]
Usually my stomach – [is still/Quiet – [Moves a lot/Is noisy]
Drink 1 was – [Very unpleasant – Very pleasant]
Drink 2 was – [Very unpleasant – Very pleasant]

***Study inclusion/exclusion criteria***
*Inclusion*

• 18-65 years old

*Exclusion*

• Epilepsy

• Stroke

• Previous or current diagnosis of schizophrenia or related conditions

• Previous or current diagnosis of bipolar disorder

• Current substance use disorder

• Pain disorder

• Current diagnosis of diabetes

• Previous cardiovascular surgery

• Cardiac pacemaker or implantable cardioverter defibrillator

• Bradycardia

Previous or current diagnosis of a significant:

• Cardiovascular condition or

• Respiratory condition

Currently taking:

• Antipsychotics

• Corticosteroids

• Opiates or opioids

• Currently taking certain antidepressants, specifically: NASSAs (e.g. mirtazapine), TCAs (e.g. amitriptyline), SARIs (e.g. trazodone), MAOIs (e.g. tranylcypromine) NOTE: if participants were taking SSRIs or SNRIs and their dose had not changed in the last month they could take part in the study.

***Nutritional contents of drinks***

*Sparkling water (Buxton®)*

|  | *Per Litre* |
| --- | --- |
| Calcium | 55mg |
| Magnesium | 19mg |
| Potassium | 1mg |
| Sodium | 24mg |
| Bicarbonate | 248mg |
| Chloride | 37mg |
| Sulphate | 13g |
| Nitrate | <0.1mg |
| Dry Recidue at 180oc | 280mg |
| pH at source | 7.4mg |

*High protein meal-replacement shake (Unsweetened Unflavoured Black Edition Huel)*

Ingredients: Pea Protein, Ground Flaxseed, Brown Rice Protein, Tapioca Starch, Sunflower Oil Powder, Medium-Chain Triglyceride Powder (from Coconut), Xanthan Gum, Potassium Citrate, Potassium Chloride, Corn Starch, Calcium Carbonate, Sodium Chloride, Green Tea Extract Powder, Kombucha Tea Powder, Ascorbic Acid, Bacillus Coagulans MTCC5856, Niacinamide, Lutein, Calcium-D-Pantothenate, Lycopene, Pyridoxine Hydrochloride, Riboflavin, Retinyl Acetate, Thiamine Mononitrate, Zeaxanthin, Menaquinone-7, L-Methylfolate, Potassium Iodide, Vitamin D2, Plant-Derived Vitamin D3, Cyanocobalamin.

***Task equipment***

***Figure. Schematic diagram of the experimental setup.*** *The digital stethoscope was connected via male audio to dual male audio cable, whereby one audio jack was plugged into the microphone jack of a USB audio adaptor plugged into the laptop running Spike2 (CED) for physiological recording, and the other audio jack into the microphone port of the task computer. Audio from the stethoscope was recorded at a sampling frequency of 1000Hz. The stethoscope settings were as follows: Filter setting = 1 [30Hz - 500Hz]; Volume = 0; Auto Shut Off Time Programming = Disabled.*

***Code availability:***

All code to run the task is available at: https://github.com/TheEmBodyStudy/RumbleRecognition. Here you will also find installation instructions, links to the equipment used, and comprehensive instructions to run the task. Code to perform the analysis in this manuscript will also be made available.
